# The impact of emotional leadership on Chinese subordinates’ work engagement: role of intrinsic motivation and traditionality

**DOI:** 10.1186/s40359-022-01022-0

**Published:** 2022-12-31

**Authors:** Jin Wan, Wenjun Zhou, Mingyue Qin, Haiming Zhou, Pingping Li

**Affiliations:** 1 East China Jiaotong University, Nanchang, 330013 China; 2 Research Centre for High Speed Railway and Regional Development, East China Jiaotong University, Nanchang, 330013 China; 3 Jiangxi Institute of Talent and Industry Integration Development, East China Jiaotong University, Nanchang, 330013 China; 4 Shandong University of Science and Technology, Taian, 271000 China

**Keywords:** Emotional leadership, Intrinsic motivation, Traditionality, Work engagement, Self-determination theory

## Abstract

**Background:**

Leaders’ emotions and emotion regulation strategies influence subordinates’ attitudes and behaviors, while previous studies have mostly taken an emotional perspective. Leaders’ emotional competence also has an impact on subordinates through motivational and cognitive pathways. Based on self-determination theory, this study examined the impact of emotional leadership on subordinates’ work engagement, as well as the mediating role of subordinates’ intrinsic motivation and the moderating role of traditionality.

**Methods:**

We first performed a scenario experiment study in which 116 Chinese college students were asked to read experimental materials on different leadership behaviors and answer relevant questions. Subsequently, a questionnaire survey was conducted, in which 347 Chinese enterprise employees were asked to rate their own experiences with emotional leadership, work engagement and intrinsic motivation. We used SPSS 25.0 for performance reliability analysis, correlation analysis and hierarchical regression analysis to test the reliability of the scales and investigate the relationship between the variables. Bootstrap analysis was used to test the mediating and moderating effects.

**Results:**

Emotional leadership has a significant direct positive effect on subordinates’ work engagement and positively influences subordinates’ work engagement through the mediation of subordinates’ intrinsic motivation. The effect of emotional leadership on intrinsic motivation is stronger for those with high traditionality than for those with low traditionality.

**Conclusion:**

Emotional leadership can improve subordinates’ work engagement by stimulating their intrinsic motivation. Therefore, managers need to be able to effectively regulate and manage subordinates’ emotions to stimulate their intrinsic motivation and to differentiate the management of subordinates with different levels of traditionality to improve subordinates’ work engagement.

## Introduction

Work engagement is a state in which an individual is full of energy, concentration and dedication at work [1] and is a key factor in improving individual effectiveness and productivity [2]. Therefore, how to promote employee work engagement is an important issue for managers. With the rise of positive psychology, scholars are focusing on the effect of subordinates’ emotions on their work engagement, such as emotional intelligence [3], depression and anxiety [4]. However, according to affective events theory, employee emotions are often triggered by external events at work [5]. In particular, leaders, as an important aspect of the work environment, often have significant impacts on subordinates’ emotions. Therefore, leaders’ emotions and emotion regulation strategies are one of the key causes of subordinates’ emotions, attitudes and behaviors [6].

Emotional leadership refers to the leadership process in which leaders effectively use their own emotional capabilities and affect the emotional perception of subordinates to improve leadership effectiveness [7]. Emotional leadership emphasizes the management and contagion of the emotions of the members of the organization. However, few studies have examined the influence of emotional leadership on subordinates’ work behavior. According to social exchange theory, when leaders are able to effectively regulate and manage subordinates’ emotions, subordinates will feel supported by leaders and will be more engaged in their work to reward the organization [8]. However, previous studies have rarely directly tested the relationship between emotional leadership and subordinates’ work engagement. Moreover, previous research has discussed the impact of leadership on subordinates’ work attitudes and behaviors from the affective perspective [9, 10], while it also affects subordinates through motivational pathways [11] by meeting their needs for autonomy, competence, and relationships [12–16], resulting in work engagement, which has rarely been investigated in the field of emotional leadership. In addition, the theory also emphasizes that employee motivation is the product of the interaction between individual characteristics and the external environment [17]. Traditionality, as an individual characteristic with a typical Chinese cultural imprint and reflecting differences in individual values, has a significant impact on employees’ behaviors [18] and may be an important moderator in the process of leadership influencing subordinates’ work engagement. However, existing research has not attempted to reveal whether the influences of emotional leadership are different on subordinates with different levels of traditionality.

Based on self-determination theory, this study investigated the influence of emotional leadership on subordinates’ work engagement and examined the role of intrinsic motivation and traditionality to enrich emotional leadership effectiveness research and provide a reference for enhancing subordinates’ work engagement.

## Literature review and hypotheses

### Emotional leadership and work engagement

In emotional leadership, the emotional competencies of leaders include emotional intelligence, empathy, emotion recognition, emotion expression and emotion regulation [7]. Studies have found that leaders with high emotional intelligence are effective in promoting employees’ engagement and performance [19, 20]. The most obvious characteristic of people with high emotional intelligence is that they are good at identifying and managing their own and others’ emotions [21]. Therefore, leaders who are able to identify and manage their own emotions and those of their subordinates can increase subordinates’ work engagement. In addition, empathy, a core component of emotional leadership [22], helps to ease the negative emotions of subordinates in certain situations and helps them re-engage in their work [16, 23].

In summary, this study proposes Hypothesis 1: Emotional leadership has a significant positive effect on work engagement.

### The mediating role of intrinsic motivation

Intrinsic motivation is an individual’s willingness to work due to an interest in the work itself [24] and is influenced by environmental factors such as organizational culture [25]. Leadership is an important environmental variable that influences employee intrinsic motivation [26]. Positive leadership styles such as ethical and Spiritual leadership have been found to promote employees’ intrinsic motivation [27–29].

Self-determination theory states that work environments can effectively stimulate individuals’ intrinsic motivation when they meet subordinates’ needs for autonomy, competence and relationships [12]. Common emotion management behaviors in emotional leadership include emotion-oriented task setting, relationship management, boosting morale, caring and support, open communication, and witty interaction [14]. On the one hand, leaders with high emotional leadership usually provide subordinates with adequate altruistic care and resource support, allow them to organize their own work schedules, and boost their confidence in completing difficult tasks by boosting morale, which will enhance their subordinates’ sense of job autonomy and competence [14, 15]; on the other hand, leaders with high emotional leadership often engage in positive emotional interactions and open communication with subordinates, which is conducive to building a harmonious relationship between supervisors and subordinate [16], which can meet the relationship needs of subordinates. Thus, emotional leadership is effective in meeting subordinates’ autonomy, competence and relationship needs, which in turn stimulate the intrinsic motivation of subordinates.

Self-determination theory further states that motivation at work is an important mediating variable between external environmental factors and individual work attitudes and behaviors [30]. Subordinates are usually more motivated to engage in work when they are interested in the work itself [13]. Therefore, intrinsic motivation is the key force that motivates individuals to engage in their work [31]. In addition, autonomy and competence, core cognitive components of intrinsic motivation [32], can arouse subjective initiative and work enthusiasm of employees, make them focus on their work, and result in significantly higher levels of work engagement [33].

In summary, this study proposes Hypothesis 2: Emotional leadership motivates subordinates’ intrinsic motivation, which in turn increases their work engagement. In other words, subordinates’ intrinsic motivation plays a mediating role between leaders’ emotional leadership and subordinates’ work engagement.

### The moderating role of subordinates’ traditionality

Traditionality has been identified and measured as the extent to which an individual endorses the traditional hierarchical role relationships (i.e., emperor-subject, father-son, husband-wife, older brother-younger brother, and friend-friend) prescribed by Confucian social ethics [34]. Deference to authority is a core component of traditionality in the workplace [35]. Individuals with high traditionality follow their role obligations and return more “loyalty” when given more “benevolence” by the leader, whereas individuals with low traditionality follow the principle of incentive-contribution equality, and their behaviors are more influenced by reciprocal exchange with the leader [35].

According to leadership contingency theory, leadership effectiveness is influenced by situational factors [36]. Subordinates respond differently to leadership behaviors depending on their traditionality [37]. First, subordinates with high traditionality follow cultural values such as humaneness, benevolence and morality, and the caring and supportive, open communication and morale-boosting characteristics of emotional leadership match their values [35, 38]. As a result, they are more likely to recognize emotional leadership and be more influenced by it. Second, subordinates with high traditionality will show more respect, obedience and admiration for their leaders [18], which leads them to show greater compliance with emotional management strategies and respond more positively to emotional leadership behaviors. Thus, they will be more susceptible to the benefits of emotional leadership and are more likely to satisfy their needs for autonomy, competence and relationships and to be internally motivated to work.

In summary, this study proposes Hypothesis 3: Traditionality positively moderates the relationship between emotional leadership and subordinates’ intrinsic motivation. The positive effect of emotional leadership on intrinsic motivation is stronger for subordinates with high traditionality.

**Fig. 1:**
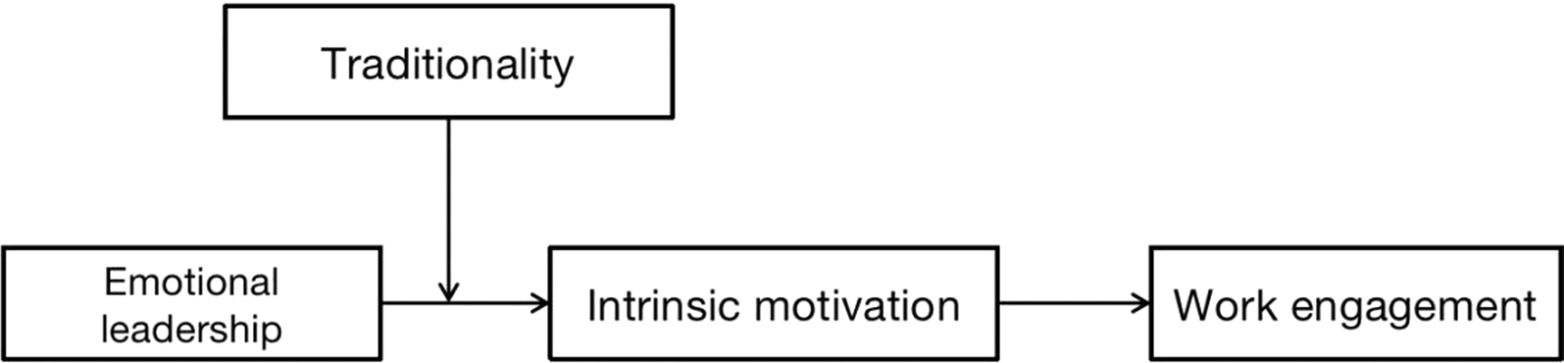
The research model

Thus, this study constructs the research model shown in Fig. 1:

## Study 1: Experimental study

### Methods

#### Experimental procedures

G*Power was used to estimate the sample size, and one-way ANOVA was selected, in which α = 0.05, 1−β = 0.80, f = 0.4, the number of groups = 4, and the total number of required samples was 111. The participants were students from a Chinese university majoring in economic management, including human resource management, accounting and finance.

They were told they would participate in a role simulation experiment on leadership behavior, which required them to read materials and answer relevant questions. A total of 126 participants were recruited. The traditionality test was carried out first, and participants were divided into high traditionality and low traditionality groups according to their scores of traditionality higher and lower than the mean. Within the two groups, participants were randomly divided into high and low emotional leadership subgroups and then read the high and low emotional leadership materials, respectively. The material of high emotional leadership described a leader who is sensitive to the emotions of subordinates, cares about their work and lives, and gives timely help and encouragement to them, while the material of low emotional leadership was just the opposite (see the appendix for specific materials). Then, they completed emotional leadership, intrinsic motivation and work engagement questionnaires.

Generally, it takes approximately 5 s to answer each question, and the minimum time for this questionnaire is approximately 105 s. Therefore, after excluding those questionnaires that took less than 100 s to complete, 116 questionnaires were valid. Of these, 88 were female and 28 were male. The final valid sample of the four groups is shown in Table 1. Specifically, there were 26 students in the high emotional leadership and high traditional group, of these, 14were female and 12 were male; 35 students in the high emotional leadership and low traditional group, of these, 31 were female and 4 were male; 21 students in the low emotional leadership and high traditional group, of these, 12 were female and 9 were male; and 34 students in the low emotional leadership and low traditional group, of these, 31 were female and 3 were male.

**Table 1:** Sample groupings

Group	High emotional leadership	Low emotional leadership
High traditionality	26	21
Low traditionality	35	34

The number in the table represents the sample size

Thirty-two university students were invited to conduct a pretest to test the validity of the text material before the formal experiment. After reading the materials, all participants rated the leaders in the material they had read using a five-point emotional leadership scale (for a detailed description of the scale, see the Measures section). The results showed that the mean score of emotional leadership of the high emotional leadership material group was significantly higher (M = 4.50, SD = 0.44) than that of the low emotional leadership material group (M = 1.75, SD = 0.66). This indicated that the two text materials could trigger significantly different perceptions of emotional leadership.

#### Measures

All scales used in this study were scored on a 5-point Likert scale, from 1 to 5 representing “totally disagree” to “totally agree”, respectively.

Emotional leadership was measured using Jin’s revised Emotional Competence Scale [22], which was commonly used in measurement work and has shown good reliability in previous studies [39]. It contains seven questions, such as, “My team leader is able to identify subordinates’ strengths and limitations”. The Cronbach’s alpha in this study was 0.98.

Intrinsic motivation was measured using the scale developed by Deci and Ryan [40], which was commonly used in measurement work and has shown good reliability in previous studies [41]. It contains four questions, such as, “I have fun doing my job”. The Cronbach’s alpha in this study was 0.98.

Traditionality was measured using the scale developed by Farh et al. [18], which was commonly used in measurement work and has shown good reliability in previous studies [42]. It contains five questions: (1) “When people have a conflict, they should ask the most senior person to decide who is right,” (2) “Children should respect those people who are respected by their parents,” (3) “The best way to avoid mistakes is to follow the instructions of senior persons,” (4) “Before marriage, a woman should subordinate herself to her father; after marriage, to her husband,” and (5) “The chief government official is like the head of a household, the citizen should obey his decisions on all state matters.” The Cronbach's alpha in this study was 0.72.

Work engagement was measured using the scale developed by Saks [43], which was commonly used in measurement work and has shown good reliability in previous studies [44]. It contains five questions such as “At work, I concentrate on my job”. The Cronbach's alpha in this study was 0.93.

#### Statistical analysis

SPSS 25.0 and Mplus 7.0 were used for statistical analysis in this study. Reliability analysis was used to test the reliability of the scales, while correlation analysis and hierarchical regression analysis were used to test the hypothesis to investigate the relationship between the independent variable emotional leadership, the mediating variable intrinsic motivation, the moderating variable traditionality and the outcome variable work engagement. To test the positive impact of emotional leadership on intrinsic motivation and the moderating role of traditionality, ANOVA was used to compare the intrinsic motivation of different groups. All scales in this study are mature scales with a single dimension, so exploratory factor analysis is not needed.

## Results

### Manipulation test

ANOVA results showed that the high emotional leadership group rated emotional leadership significantly higher (M = 4.03, SD = 0.66) than the low emotional leadership group (M = 1.84, SD = 0.71), *t*(116) = 17.09, *p* < 0.001. Therefore, this experiment was effective in manipulating emotional leadership.

#### Correlation analysis

As shown in Table 2, emotional leadership was significantly and positively correlated with intrinsic motivation (r = 0.90, *p* < 0.01) and work engagement (r = 0.78, *p* < 0.01); and intrinsic motivation was significantly and positively correlated with work engagement (r = 0.84, *p* < 0.01). The hypotheses were initially tested.

**Table 2:** Table of correlation coefficients for the study variables

Variables	M	SD	Emotional leadership	Intrinsic motivation	Traditionality	Work engagement
Emotional Leadership	2.99	1.29				
Intrinsic motivation	2.91	1.27	0.90^**^			
Traditionality	2.81	0.64	0.08	0.08		
Work engagement	2.75	0.92	0.78^**^	0.84^**^	0.14	

*M* mean; *SD* standard deviation

**correlation significant at the 0.01 level (two-tailed)

#### Hypothesis testing

Hierarchical regression analysis was used to test the hypothesis, and the results are shown in Table 3. To explore the relationship between emotional leadership and work engagement, emotional leadership was added to obtain M3. In M3, the positive effect of emotional leadership on work engagement was significant (*β* = 0.78, *p* < 0.001); thus, Hypothesis 1 was supported. To explore the relationship between emotional leadership and intrinsic motivation, emotional leadership was added to obtain M1. In M1, the positive effect of emotional leadership on intrinsic motivation was significant (*β* = 0.90, *p* < 0.001). To explore the mediating effect of intrinsic motivation, on the basis of M3, intrinsic motivation was added to obtain M4. In M4, the positive effect of intrinsic motivation level on work engagement was significant (*β* = 0.76, *p* < 0.001), and the β coefficient of emotional leadership on work engagement decreased from 0.78 to 0.10 and was no longer significant. Thus, Hypothesis 2 was supported. To explore the moderating effect of traditionality, an interaction term of emotional leadership and traditionality was constructed. Then, on the basis of M1, the interaction term was added to obtain M2. In M2, the interaction term between emotional leadership and traditionality had a significant positive effect on intrinsic motivation (*β* = 0.09, *p* < 0.05); thus, Hypothesis 3 was supported.

**Table 3:** Hierarchical regression analysis table

	Intrinsic motivation				Work engagement				
	M1		M2		M3			M4	
	*β*	*SE*	*β*	*SE*	*β*	*SE*	*β*		*SE*
Emotional leadership	0.90^***^	0.04	0.92^***^	0.04	0.78^***^	0.042	0.10		0.09
Intrinsic motivation							0.76^***^		0.09
Traditionality			− 0.01	0.08					
Emotional leadership* traditionality			0.09^*^	0.06					
R^2^	81%		82%		61%		71%		
△R^2^	81%		1%		61%		10%		

M1, Model 1; M2, Model 2; M3, Model 3; M4, Model 4; SE, standard errors; *p < 0.05; ***p < 0.001

**Fig. 2:**
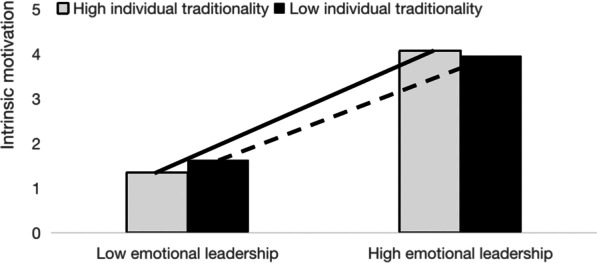
The moderating role of traditionality in the relationship between emotional leadership and intrinsic motivation

To provide a more visual representation of the moderating effect of traditionality, ANOVA was used to compare the intrinsic motivation scores of the four groups of subjects, and the results are shown in Fig. 2. Within the low traditionality group, the mean score of intrinsic motivation of the low emotional leadership subgroup (M = 1.91, SD = 0.86) was significantly lower than that of the high emotional leadership subgroup (M = 3.90, SD = 0.67) (*p* < 0.001). Within the high traditionality group, the mean score of intrinsic motivation of the low emotional leadership subgroup (M = 1.67, SD = 0.60) was also significantly lower than that of the high emotional leadership subgroup (M = 4.03, SD = 0.42) (*p* < 0.001). However, the positive effect of emotional leadership on intrinsic motivation was also stronger when the level of traditionality increased, suggesting that traditionality positively moderates the relationship between emotional leadership and intrinsic motivation; thus, Hypothesis 3 was supported.

## Study 2: Questionnaire study

### Methods

#### Participants

According to the requirement of 10 samples for each parameter to be estimated, this study required nearly 250 samples. According to the 60% efficiency of the distributed questionnaires, 400 questionnaires were initially distributed to a large science and technology enterprise in Beijing, China. The company specializes in R&D and the production of mining machinery and equipment and has more than 500 employees. Participants who had been working there for more than 6 months were included in the survey, and 382 questionnaires were returned. Unlike Study 1, there was no experimental manipulation, and employees were asked to rate their own experiences for all questionnaires. After excluding 90% or more of the data that only selected the same option, the filling rate was less than 80%, and the data exceeded 3 standard deviations, a total of 347 valid questionnaires were obtained.

Among respondents, 71.8% were female and 28.2% were male; those aged 25 and below accounted for 15.6%, those aged 26–30 accounted for 54.7%, those aged 31–35 accounted for 20.5%, and those aged 36 and above accounted for 9.2%; 31.7% had junior secondary education or below, 43.8% had high school or secondary education, 19.6% had a college education and 4.9% had a bachelor's degree or above, and 68.0% had worked under their current supervisor for less than 3 years.

#### Measures

All scales used in this study were scored on a 5-point Likert scale, from 1 to 5 representing “totally disagree” to “totally agree”, respectively. Emotional leadership, internal motivation and traditionality were all measured using the same scales as in Study 1, with Cronbach's alphas of 0.90, 0.92 and 0.67, respectively. Work engagement was measured using the scale developed by Schaufeli [45], which was commonly used in measurement work and has shown good reliability in previous studies [46]. It contains nine questions such as “At my work, I feel bursting with energy”. The Cronbach's alpha in this study was 0.91.

#### Statistical analysis

SPSS 25.0 and Mplus 7.0 were used for statistical analysis in this study. Reliability analysis and confirmatory factor analysis were used to test the reliability and validity of the scales, and common method bias factor analysis was used to test whether the data had homology bias problems. Correlation analysis and hierarchical regression analysis were used to test the hypothesis to investigate the relationship between the independent variable emotional leadership, the mediating variable intrinsic motivation, the moderating variable traditionality and the outcome variable work engagement. Finally, bootstrap analysis was performed using the Process plugin to integrate the research model and to analyze the mediating role of intrinsic motivation between emotional leadership and work engagement and the moderating role of traditionality between emotional leadership and intrinsic motivation.

### Results

#### Discriminant validity analysis

Confirmatory factor analysis was performed on all variables in the research model using Mplus7.0 to test the discriminant validity between variables. This study compared the fit of models with one to four factors. As shown in Table 4, the fitting index of the four-factor model was significantly better than that of the one-to-three-factor models, and the fitting indices of the four-factor model all met the discriminant criteria, indicating that the four research variables had good discriminant validity, which can also be further verified from the low correlation among variables in the following correlation analysis.

**Table 4:** Confirmatory factor analysis results of variables

	χ^2^	*df*	χ^2^/*df*	RMSEA	SRMR	TLI	CFI
Four factors	542.60	269	2.02	0.05	0.04	0.94	0.95
Three factors	749.43	272	2.76	0.07	0.06	0.90	0.91
Two factors	956.27	274	3.49	0.08	0.06	0.85	0.87
One factor	1863.59	275	6.78	0.13	0.11	0.66	0.69

*N* = 347; the three-factor model takes intrinsic motivation and traditionality as a factor; the two-factor model takes intrinsic motivation, traditionality and work engagement as a factor

#### Common method bias test

Common method bias refers to the artificial variation among variables caused by the same subjects or data sources, measurement context, project context, or the characteristics of the project itself. Considering that all the data were self-reported by the research subjects at one time, to reduce the impact of common method bias on the research results, the following analysis was conducted. First, the Harman one-way test for common method bias was adopted, and all variable items were subjected to unrotated exploratory factor analysis. The percentage of variance explained by the first factor was 38.446%, which was lower than the 40% criterion. Second, since the Harman one-way test method may be insensitive, the method factor was added as a global factor on the basis of the four-factor model. The four-factor structure of the data fit well (χ^2^/*df* = 2.02, CFI = 0.95, TLI = 0.94, SRMR = 0.04, RMESA = 0.05), but the model could not be fitted after adding the method factor. Finally, positive team climate was used as a marker variable that had no theoretical relationship with this study. The model after adding the marker variable (BIC = 21,230.77) was compared with the research model (BIC = 19,254.31), and the research model was significantly better than the model with a marker variable. All the above statistical tests showed that there was no serious common method bias in these data [47].

#### Correlation analysis

Table 1 presents the means, standard deviations, and correlations. As shown in Table 5, there were correlations between variables such as gender, age, marriage, education, coworking time and frequency interaction and research variables, including emotional leadership, intrinsic motivation, work engagement and traditionality. Therefore, the former variables were treated as control variables in this study.

**Table 5:** Descriptive statistics and correlations

Variables	M	SD	1	2	3	4	5	6	7	8	9	10
1.Gender	1.28	0.45										
2.Age	29.33	4.61	− 0.04									
3.Marriage	1.54	0.51	− 0.11^*^	0.51^**^								
4.Education	1.99	0.89	− 0.10	− 0.04	0.14^**^							
5.Coworking time	3.10	2.45	0.01	0.34^**^	0.32^**^	0.09						
6.Frequency of interaction	3.21	1.12	− 0.01	0.03	0.02	− 0.08	0.09					
7.Emotional Leadership	3.56	0.71	− 0.05	− 0.07	− 0.06	0.14^*^	− 0.06	0.30^**^				
8.Intrinsic motivation	3.42	0.80	− 0.04	0.06	0.05	0.16^**^	0.10	0.20^**^	0.41^**^			
9.Work engagement	3.39	0.67	− 0.08	0.02	0.03	0.15^**^	0.08	0.19^**^	0.48^**^	0.78^**^		
10.Traditionality	3.09	0.62	− 0.16^*^	− 0.09	0.03	0.25^**^	0.08	0.04	0.18^**^	0.28^**^	0.31^**^	

*M* mean; *SD* standard deviationa

*correlation significant at the 0.05 level (two-tailed).

**correlation significant at the 0.01 level (two-tailed)

Emotional leadership was significantly and positively correlated with intrinsic motivation (r = 0.41, *p* < 0.01) and with work engagement (r = 0.48, *p* < 0.01); intrinsic motivation was significantly and positively correlated with work engagement (r = 0.78, *p* < 0.01). The hypotheses were initially tested.

#### Hypothesis test

As shown in Table 6, each hypothesis was tested using hierarchical regression analysis.

**Table 6:** Hierarchical regression analysis

Control variables	Intrinsic motivation						Work engagement					
	M1		M2		M3		M4		M5		M6	
	*β*	SE	*β*	SE	*β*	SE	*β*	SE	*β*	SE	*β*	SE
Gender	− 0.02	0.09	− 0.01	0.08	0.02	0.08	− 0.06	0.08	− 0.05	0.07	− 0.05	0.05
Age	0.05	0.01	0.06	0.01	0.09	0.01	0.02	0.01	0.03	0.01	− 0.01	0.01
Marriage	− 0.02	0.10	0.01	0.09	0.01	0.09	− 0.04	0.08	− 0.01	0.07	− 0.01	0.05
Education	0.18^**^	0.05	0.11^*^	0.05	0.05	0.04	0.16^**^	0.04	0.08	0.04	0.01	0.03
Coworking time	0.06	0.02	0.09	0.02	0.07	0.02	0.05	0.02	0.09	0.01	0.03	0.01
Frequency of interaction	0.21^**^	0.04	0.09	0.04	0.09	0.04	0.20^**^	0.03	0.05	0.03	− 0.01	0.02
*Independent variable*												
Emotional leadership			0.38^**^	0.06	0.36^**^	0.06			0.46^**^	0.05	0.19^**^	0.04
*Mediate variable*												
Intrinsic motivation											0.70^**^	0.03
*Moderate variable*												
Traditionality					0.16^**^	0.06						
Emotional Leadership* Traditionality					0.15^**^	0.08						
R^2^	8%		21%		26%		7%		25%		64%	
△R^2^	8%		13%		5%		7%		18%		39%	

M1, Model 1; M2, Model 2; M3, Model 3; M4, Model 4; M5, Model 5; M6, Model 6; SE, standard errors; *p < 0.05; **p < 0.01

Main effect tests. In M4, only the control variables are added to the regression equation. To explore the relationship between emotional leadership and work engagement, based on M4, emotional leadership was added to obtain M5. In M5, emotional leadership showed a significant positive effect on employees’ work engagement (*β* = 0.46, *p* < 0.01), indicating that the higher the level of emotional leadership of the leader, the higher the level of employee work engagement; thus, Hypothesis 1 was supported.

Mediating effect test. In M1, only the control variables are added to the regression equation. To explore the relationship between emotional leadership and intrinsic motivation, based on M1, emotional leadership was added to obtain M2. In M2, the positive effect of emotional leadership on employees’ intrinsic motivation was significant (*β* = 0.38, *p* < 0.01). To explore the mediating effect of intrinsic motivation, based on M5, intrinsic motivation was added to obtain M6. In M6, the positive effect of employees’ intrinsic motivation on work engagement was significant (*β* = 0.70, *p* < 0.01), but the β coefficient of emotional leadership on employee work engagement decreased from 0.38 to 0.19, indicating that employee’s intrinsic motivation partially mediates the relationship between emotional leadership and work engagement; thus, Hypothesis 2 was supported.

Moderating effect test. To explore the moderating effect of traditionality, an interaction term of emotional leadership and traditionality was constructed. Then, on the basis of M2, the interaction term was added to obtain M3. In M3, the interaction term between emotional leadership and employee traditionality had a significant positive effect on intrinsic motivation (*β* = 0.15, *p* < 0.01) after considering the control variables.

#### Bootstrap test

**Fig. 3:**
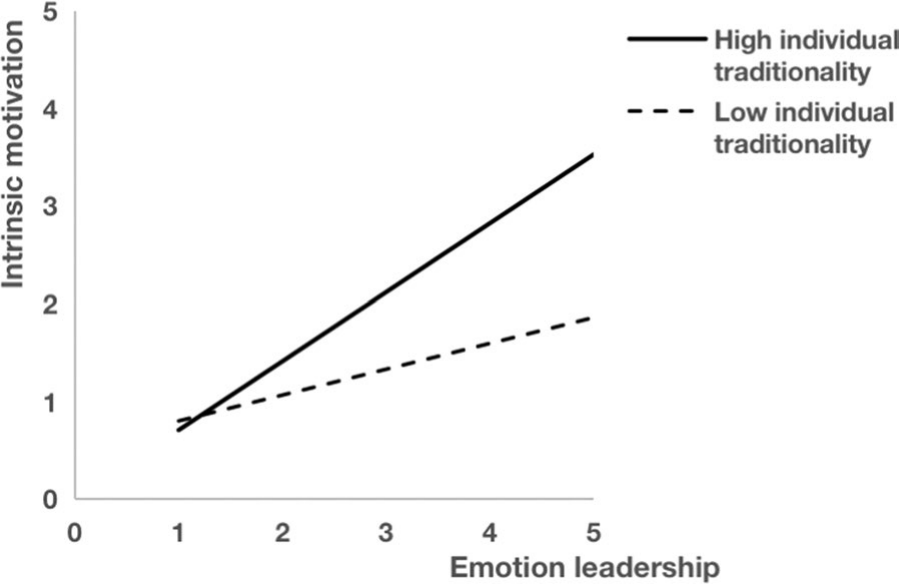
The moderating effect of traditionality

The theoretical model was then tested using the Process plug-in of SPSS. Model 7 was chosen, and 5,000 bootstrap sample analyses revealed a significant mediating effect of intrinsic motivation between emotional leadership and work engagement (*β* = 0.28, CI [0.20, 0.37]). Hypothesis 2 again was supported. Furthermore, there was traditionality regulation of the relationship between emotional leadership and intrinsic motivation (*β* = 0.22, CI [0.06, 0.37]). Simple slope analysis was adopted to analyze the moderating role of traditionality between emotional leadership and employees’ intrinsic motivation. Divide high and low groups by the mean of traditionality plus or minus one standard deviation. The results are shown in Fig. 3. In both the high and low traditionality groups, emotional leadership had a positive effect on intrinsic motivation; however, the positive effect of emotional leadership on intrinsic motivation was significantly stronger in the high traditionality group (*β* = 0.59, *p* < 0.001) than in the low traditionality group (*β* = 0.27, *p* < 0.01); as emotional leadership increased, the gap between the two groups' intrinsic motivation continued to widen. This suggested that subordinates' traditionality can enhance the effect of emotional leadership on subordinates' intrinsic motivation. Hypothesis 3 was supported.

However, we found that traditionality did not moderate the mediating effect of intrinsic motivation on emotional leadership and work engagement (*p* > 0.05).

## Discussion

First, when addressing Hypothesis 1, we found that emotional leadership contributes to improving subordinates’ work engagement, which helps to extend extant knowledge on antecedents of subordinates’ work engagement. Previous studies have mostly focused on the impact of subordinates’ own emotions on their work engagement [3, 4]. However, as an important work environment factor that affects subordinates’ emotions, the impact of leaders' emotions and emotion management strategies on subordinates' work engagement should not be ignored. This study found that emotional leadership, a more specific type of leadership that emphasizes the management and contagion of the emotions of the members of the organization, is an important factor in promoting employee engagement. When leaders effectively manage their own emotions and those of their subordinates and show care and support to their subordinates, subordinates are more engaged in their work and reward the organization. This confirms the insight that “leadership emotions are a deeper factor in employee engagement than employee emotions” [6].

Second, when addressing Hypothesis 2, we found that subordinates' intrinsic motivation plays a mediating role between leaders’ emotional leadership and subordinates' work engagement, which helps to enrich the understanding of the mechanisms by which emotional leadership affects employee engagement by introducing intrinsic motivation as a 'bridge' between them. Most previous research has been conducted from the perspective of emotional contagion, suggesting that leadership styles can change subordinates' work status by influencing their emotions [9, 10]. Based on self-determination theory, this study found that emotional leadership promotes subordinates' work engagement not only by enhancing their positive emotions but also by stimulating their intrinsic motivation to work by satisfying their needs for autonomy, competence and relationships. The relationship between leadership emotional competence and employee work engagement was discussed from a motivational perspective beyond emotional contagion, enriching the knowledge of the mechanisms by which emotional leadership affects employee engagement.

Finally, when addressing Hypothesis 3, we found that traditionality positively moderates the relationship between emotional leadership and subordinates' intrinsic motivation, which helps to explore the boundary conditions under which emotional leadership affects subordinates' motivation. Emotional leadership had a greater effect on intrinsic motivation for those with high traditionality. This echoes the call that individual differences in characteristics should be taken into account when discussing subordinates' processing of emotional information about leadership [17]. In addition, values from different cultural backgrounds influence individuals’ interpretations of situations, which in turn influence their psychological and behavioral responses [48]. Traditionality is the most representative value of Chinese people. This study confirms the moderating role of traditionality in the relationship between emotional leadership and subordinates' intrinsic motivation, which validates the influence of different cultural values on subordinates' motivation and contributes to the contextualization of management theory.

### Practical implication

Based on the above findings, the following management implications emerge from this study.

First, when selecting leaders, organizations should identify their level of emotional leadership and strengthen managers' leadership development in self-emotion management and empathy skills. Furthermore, when assigning work tasks and setting work goals, managers should consider employees’ emotional responses, so it is best to match subordinates' abilities as much as possible so that they can successfully complete their tasks; when subordinates face difficulties, managers should help them identify their own emotions and provide emotional and informational support at the right time. Leaders can infect subordinates' emotions through public appreciation and humor and attach importance to fairness in interpersonal interactions and get along with each employee in a fair and equal manner.

Second, managers should stimulate subordinates' intrinsic motivation by meeting subordinates' autonomy, competence and relationship needs in their daily management. For example, participatory management, delegated management or management by objectives should be adopted for mature subordinates. When employees perform well, leaders should commend them in a timely manner to strengthen their sense of achievement, encourage them in their daily work, and make employees feel recognized; they could also give employees personalized care and create a respectful and trusting leader-member relationship.

Finally, it is relatively difficult to change individual traditionality to increase the effect of emotional leadership on subordinates’ intrinsic motivation. Therefore, it is important for managers to first identify subordinates’ degree of traditionality and to differentiate the management of subordinates with different levels of traditionality. Managers should focus more on the management of subordinates with low traditionality, such as communicating regularly with them and providing more care and support and extrinsic motivation.

### Limitations and future directions

There are some limitations to this study.

First, the variables measured in this study were all self-reported by the subjects at one time point. Although statistical tests indicated no significant common method bias, future research should invite supervisors to evaluate the work engagement of their subordinates and encourage subordinates to evaluate the emotional leadership of their supervisors. This study was a cross-sectional study, which does not reflect well the causal relationship between variables. A longitudinal study design should be adopted to verify the causal relationship between variables, which has been tested in experimental Study 1. In addition, the samples in the experimental study are college students, and the assessment of work engagement is related to the students’ imaginary responses. In the future, employees can be selected to conduct experiments to further verify the credibility of the research results.

Second, this study explored the impact of emotional leadership on subordinates' work engagement from self-determination theory; more mechanisms of emotional leadership can be explored from the perspective of other theories in the future. For example, based on job demand-resource theory, future studies can explore whether emotional leadership can influence subordinates' work engagement by enhancing their psychological resources under high work demands. Based on the cognitive-affective processing system, we could also introduce both cognitive and affective pathways to compare through which pathway emotional leadership has a greater influence on subordinates’ work engagement.

Finally, this study only discussed the moderating role of traditionality, while the moderating role of other work values, such as career centrality and power distance orientation, could also be considered in the future.

## Conclusions

Based on self-determination theory, this study not only revealed the impact of emotional leadership on subordinates' work engagement, extending the research on the leadership antecedents of subordinates' work engagement and supporting the insight that leadership emotions are an important factor in employee engagement but also took intrinsic motivation as a bridge connecting them and clarifying the motivation path of emotional leadership on work engagement. However, this study found that intrinsic motivation only partially mediated the effect of emotional leadership on work engagement, and other pathways of emotional leadership's impact on work engagement could be explored in the future. Moreover, it introduced traditionality as a boundary condition and found that emotional leadership has different effects on intrinsic motivation for subordinates with different personalities, echoing the call that individual differences in characteristics should be taken into account when discussing subordinates' processing of emotional information about leadership. Future research could further explore the moderating role of variables such as subordinate power distance to enrich the understanding of the boundary conditions of their relationship. Finally, this study showed that it is necessary to cultivate leaders' emotional leadership and stimulate subordinates' intrinsic motivation. Leaders should carry out differentiated management for employees with different levels of traditionality to improve subordinates' work engagement.

## Data Availability

The raw data supporting the conclusions of this article will be made available by the authors, without undue reservation. If someone wants to request the data, they can contact 734,141,500@qq.com.

## Appendix

High emotional leadership material is described as follows: "Your performance has been poor this month, and Mr. Chen, the supervisor, is keenly aware of the problem. During teatime yesterday, he came up to you and asked if you were experiencing any difficulties in your life. When he knew that you needed to take care of your seriously ill father every day after work recently, he took the initiative to introduce you to his friend, a doctor in the specialty; when he heard that you felt the job he assigned you was suitable, but the workload was slightly heavy, he patiently asked you if you needed to adjust your work and pointed out your advantages. He then provided you with suggestions to improve work efficiency. When he heard that your colleague, Xu Lei, was not cooperating with you at work, he encouraged you to actively communicate with him and pointed out the advantages of Xu Lei, helping you understand how to work with Xu Lei. During the chat, he kept expressing his belief that you would do better. You felt very happy chatting with him and felt confident about your future after the chat. You mentioned to him the problem of the short lunch break yesterday. At the regular meeting today, he organized everyone to discuss this issue. In the end, after considering everyone's opinions, he decided to extend the lunch break by 15 min."

Low emotional leadership material is described as follows: "Your performance has been poor this month, and your supervisor, Mr. Chan, criticized you in front of your colleagues in the department at the regular departmental meeting. Even though you felt hurt, you still took the initiative to communicate with him and to listen to his suggestions. He did not realize that you wanted to seek his help, did not give you the opportunity to express yourself, but subjectively attributed your poor performance to three reasons. First, he criticized you for always leaving work on time and not spending enough time at work, but forgot that you specifically told him you need to take care of your seriously ill father every day after work recently. Second, he pointed out that you are not competent enough. However, you had communicated to him several times that the work assigned to you was not within your expertise, but he never adjusted your work. Third, he pointed out that you did not cooperate well with colleague Xu Lei, but he ignored that Xu Lei is a maverick and cannot get along with anyone in your team. In the process of communication, he thought he understood what you were trying to express, but you felt unappreciated and the confusion was left unresolved. The chat broke up unhappily."

## References

[1] Schaufeli WB, Salanova M, González-Romá V (2002). The measurement of engagement and burnout: a two sample confirmatory factor analytic approach. J Happiness Stud.

[2] Ozturk A, Karatepe OM, Okumus F (2021). The effect of servant leadership on hotel employees’ behavioral consequences: work engagement versus job satisfaction. Int J Hosp Manag.

[3] Wang L (2021). Exploring the relationship among teacher emotional intelligence, work engagement, teacher self-efficacy, and student academic achievement: a moderated mediation model. Front Psychol.

[4] Hentrich S, Zimber A, Sosnowsky-Waschek N (2017). The role of core self-evaluations in explaining depression and work engagement among managers. Curr Psychol.

[5] Ashkanasy NM, Dorris AD (2017). Emotions in the workplace. Ann Rev Organ Psychol Organ Behav.

[6] Khan H, Chowdhury MS, Kang D (2022). Leaders’ emotion regulation and the influence of respect and entitlement on employee silence. Sustainability.

[7] Peng J, Liu Y, Lu H (2014). Emotional leadership: conceptualization, measurement, development and model construction. Adv Psychol Sci.

[8] Truong TVT, Nguyen HV, Phan MCT (2021). Influences of job demands, job resources, personal resources, and coworkers support on work engagement and creativity. J Asian Finance Econ Bus.

[9] Qin P, Liu Y (2019). The empirical research of the influence of leadership positive emotion on counterproductive work behavior. Psychology.

[10] Park IJ, Shim SH, Hai S (2021). Cool down emotion, don’t be fickle! The role of paradoxical leadership in the relationship between emotional stability and creativity. Int J Hum Resour Manag.

[11] Al-Sada M, Al-Esmael B, Faisal MN (2017). Influence of organizational culture and leadership style on employee satisfaction, commitment and motivation in the educational sector in Qatar. EuroMed J Bus.

[12] Deci EL, Ryan RM (1985). The general causality orientations scale: self-determination in personality. J Res Personal.

[13] Putra ED, Cho S, Liu J (2017). Extrinsic and intrinsic motivation on work engagement in the hospitality industry: test of motivation crowding theory. Tour Hosp Res.

[14] Kaplan S, Cortina J, Ruark G (2014). The role of organizational leaders in employee emotion management: a theoretical model. Leadersh Q.

[15] Martela F, Gómez M, Unanue W (2021). What makes work meaningful? Longitudinal evidence for the importance of autonomy and beneficence for meaningful work. J Vocat Behav.

[16] Toegel G, Kilduff M, Anand N (2013). Emotion helping by managers: an emergent understanding of discrepant role expectations and outcomes. Acad Manag J.

[17] Deci EL, Olafsen AH, Ryan RM (2017). Self-determination theory in work organizations: the state of a science. Annu Rev Organ Psychol Organ Behav.

[18] Farh JL, Earley PC, Lin SC (1997). Impetus for action: a cultural analysis of justice and organizational citizenship behavior in Chinese society. Adm Sci Q.

[19] Milhem M, Muda H, Ahmed K (2019). The effect of perceived transformational leadership style on employee engagement: The mediating effect of leader’s emotional intelligence. Found Manag.

[20] Zhang Y, Zhang L, Zhu J (2020). Group leader emotional intelligence and group performance: a multilevel perspective. Asian Bus Manag.

[21] MacCann C, Erbas Y, Dejonckheere E (2020). Emotional intelligence relates to emotions, emotion dynamics, and emotion complexity: a meta-analysis and experience sampling study. Eur J Psychol Assess.

[22] Jin Y (2010). Emotional leadership as a key dimension of public relations leadership: a national survey of public relations leaders. J Public Relat Res.

[23] Lin SP, Wang YY, Hsu WL (2015). The mediation effect of emotional experience between emotion labor and job engagement. Univers J Manag.

[24] Zhang Y, Liu SM (2022). Balancing employees’ extrinsic requirements and intrinsic motivation: a paradoxical leader behaviour perspective. Eur Manag J.

[25] Nguyen TM (2019). Do extrinsic motivation and organisational culture additively strengthen intrinsic motivation in online knowledge sharing? An empirical study. VINE J Inf Knowl Manag Syst.

[26] Wuisang JRH, Rawung SS, Kaligis JN (2020). The relationship between leadership and employees’ work motivation in regional secretariat office of Minahasa Regency. Society.

[27] Feng J, Zhang Y, Liu X (2018). Just the right amount of ethics inspires creativity: a cross-level investigation of ethical leadership, intrinsic motivation, and employee creativity. J Bus Eth.

[28] Shareef RA, Atan T (2018). The influence of ethical leadership on academic employees’ organizational citizenship behavior and turnover intention: mediating role of intrinsic motivation. Manag Decis.

[29] Wang M, Guo T, Ni Y (2019). The effect of spiritual leadership on employee effectiveness: an intrinsic motivation perspective. Front Psychol.

[30] Zhang J, Zhang JB, Li Y (2010). An effective path for promoting work motivation: the self-determination theory perspective. Adv Psychol Sci.

[31] Renard M, Snelgar RJ (2018). Can non-profit employees’ internal desires to work be quantified? Validating the intrinsic work motivation scale. South Afr J Psychol.

[32] Chen Z, Wu H (2008). Intrinsic motivation and its antecedents. Adv Psychol Sci.

[33] Shkoler O, Kimura T (2020). How does work motivation impact employees’ investment at work and their job engagement? A moderated-moderation perspective through an international lens. Front Psychol.

[34] Wu LZ, Liu J, Liu G (2009). Abusive supervision and employee performance: mechanisms of traditionality and trust. Acta Psychol Sin.

[35] Farh JL, Hackett RD, Liang J (2007). Individual-level cultural values as moderators of perceived organizational support–employee outcome relationships in China: comparing the effects of power distance and traditionality. Acad Manag J.

[36] Fiedler FE (1978). The contingency model and the dynamics of the leadership process. Adv Exp Soc Psychol.

[37] Yao Z, Zhang X, Liu Z (2019). Narcissistic leadership and voice behavior: the role of job stress, traditionality, and trust in leaders. Chin Manag Stud.

[38] Wang AC, Cheng BS (2010). When does benevolent leadership lead to creativity? The moderating role of creative role identity and job autonomy. J Organ Behav.

[39] Wan J, Pan TK, Peng Y (2022). The impact of emotional leadership on subordinates’ job performance: mediation of positive emotions and moderation of susceptibility to positive emotions. Front Psychol.

[40] Deci EL, Ryan RM (2013). Intrinsic motivation and self-determination in human behavior.

[41] Jeno LM, Adachi PJC, Grytnes JA (2019). The effects of m-learning on motivation, achievement and well-being: a self-determination theory approach. Br J Educ Technol.

[42] Wu Q, Zhang X, He F (2020). The multi-level influence of leader’s prosocial orientation on employee’s organizational citizenship behavior. Chin J Manag.

[43] Saks AM (2006). Antecedents and consequences of employee engagement. J Manag Psychol.

[44] Saks AM (2019). Antecedents and consequences of employee engagement revisited. J Organ Eff.

[45] Schaufeli WB, Bakker AB, Salanova M (2006). The measurement of work engagement with a short questionnaire: a cross-national study. Educ Psychol Meas.

[46] Tang HY, Long LR, Zhou RY (2015). Humble leadership behavior and subordinates’ work engagement: a mediated moderation model. J Manag Sci.

[47] Richardson HA, Simmering MJ, Sturman MC (2009). A tale of three perspectives: examining post hoc statistical techniques for detection and correction of common method variance. Organ Res Methods.

[48] Kim SH, Kim MS, Holland S (2018). Hospitality employees’ citizenship behavior: the moderating role of cultural values. Int J Contemp Hosp Manag.

